# Impact of the COVID-19 pandemic on the prevalence of respiratory viral pathogens in patients with acute respiratory infection in Shanghai, China

**DOI:** 10.3389/fpubh.2024.1230139

**Published:** 2024-02-07

**Authors:** Lifeng Pan, Yang Yuan, Qiqi Cui, Xuechun Zhang, Yujia Huo, Qing Liu, Wenwei Zou, Bing Zhao, Lipeng Hao

**Affiliations:** ^1^Shanghai Pudong New Area Center for Disease Control and Prevention, Shanghai, China; ^2^Research Base of Key Laboratory of Surveillance and Early-warning on Infectious Disease in China CDC, Shanghai, China

**Keywords:** COVID-19, acute respiratory infection, nonpharmaceutical interventions, viral pathogen, Shanghai

## Abstract

**Objective:**

This study aimed to evaluate the impact of nonpharmaceutical interventions (NPIs) taken to combat COVID-19 on the prevalence of respiratory viruses (RVs) of acute respiratory infections (ARIs) in Shanghai.

**Methods:**

Samples from ARI patients were collected and screened for 17 respiratory viral pathogens using TagMan low density microfluidic chip technology in Shanghai from January 2019 to December 2020. Pathogen data were analyzed to assess changes in acute respiratory infections between 2019 and 2020.

**Results:**

A total of 2,744 patients were enrolled, including 1,710 and 1,034 in 2019 and 2020, respectively. The total detection rate of RVs decreased by 149.74% in 2020. However, detection rates for human respiratory syncytial virus B (RSVB), human coronavirus 229E (HCoV229E), human coronavirus NL63 (HCoVNL63), and human parainfluenza virus 3 (HPIV3) increased by 91.89, 58.33, 44.68 and 24.29%, in 2020. The increased positive rates of RSVB, HPIV3, resulted in more outpatients in 2020 than in 2019. IFV detection rates declined dramatically across gender, age groups, and seasons in 2020.

**Conclusion:**

NPIs taken to eliminate COVID-19 had an impact on the prevalence of respiratory viral pathogens, especially the IFVs in the early phases of the pandemic. Partial respiratory viruses resurged with the lifting of NPIs, leading to an increase in ARIs infection.

## Introduction

Acute respiratory infections (ARIs) are common with significant morbidity and mortality worldwide, causing more than 2.5 million deaths in 2017 (1). Respiratory viruses (RVs), particularly influenza virus, coronavirus, parainfluenzavirus, adenovirus, respiratory syncytial virus, human metapneumovirus, bocavirus, rhinovirus, and enterovirus are known as common viral pathogens causing ARIs (2–10). In late 2019, a new respiratory virus, severe acute respiratory syndrome coronavirus 2 (SARS-CoV-2), emerged and caused more than 2.4 million deaths within a year (11).

Nonpharmaceutical interventions (NPIs) (12) were used to prevent the spread of SARS-CoV-2 nationwide at the beginning of COVID-19 in China, including travel restrictions, physical distancing measures and wearing masks. However, socialdistancing, masking, school closures, shelter-in-place, travel restrictions, etc^.^ (13), also changed the prevalent characteristics of ARIs caused by RVs.

Since 2011, surveillance of RVs has begun with influenza-like illness (ILI) in outpatients (14) and ARI cases (15) in Shanghai. In China, the outbreak of COVID-19 has been quickly contained, but the impact of NPIs on ARIs is still unclear. The research was conducted to investigate and evaluate whether NPI could change the etiological characteristics of ARIs in patients before and during the COVID-19 epidemic in Shanghai between 2019 and 2020.

## Materials and methods

### Ethics statement

Data on participants in this study were collected from the respiratory surveillance system of Pudong New Area Center for Disease Control and Prevention. Participants gave their verbal informed consent before collecting their information and samples. Samples and information were not collected if refused. No written consent was obtained and no measures were taken to document the process as the data would be analyzed anonymously. All information was input in the respiratory surveillance system after collection. Pursuant to the Helsinki Declaration of 1975, the study protocol and consent procedure were approved by Pudong Centre for Disease Control and Prevention Ethics Review Committee.

### Patients and specimen collection

Patients, judged by clinicians, had at least one of the following symptoms: cough, sore throat, runny nose or shortness of breath, with or without fever, were considered eligible for ARI (15). Respiratory samples (nasopharyngeal swab or sputum) and clinical data were collected and recorded by attending physicians of patients with ARI in 9 hospitals in Shanghai between January 2019 and December 2020 according to the ARI surveillance plan. The specimen was stored in 3.5-mL Viral Transport Media (VTM^™^, Yocon, Cat No: MT0301-1, Beijing, China) and transported to Shanghai Pudong New Area Center for Disease Control and Prevention for pathogen screening.

### Screening for pathogens

Suspension of VTM was used directly for nucleic acid extraction. MagNA Pure 96 DNA and Viral NA Small Volume Kit (Roche Diagnostics, Cat. NO: 06543588001, Mannheim, Germany) were used to extract the nucleic acid on MagNA Pure 96 System (Roche Diagnostics, Switzerland) according to the manufacturer’s instructions. The input volume was 200 μL and the elution volume was set at 100 μL.

Pathogens, including Adenovirus (HAdV), Human Coronavirus 229E (HCoV229E), Human Coronavirus HKU1 (HCoVHKU1), Human Coronavirus NL63 (HCoVNL63), Human Coronavirus OC43 (HCoVOC43), Human Enterovirus (EV), Influenza A/H1-2009 (IFVA-H1), Influenza A/H3 (IFVA-H3), Influenza A (IFVA), Influenza B (IFVB), Human Bocavirus (HBoV), Human Metapneumovirus (HMPV), Human Parainfluenza virus 1 (HPIV1), Human Parainfluenza virus 2 (HPIV2), Human Parainfluenza virus 3 (HPIV3), Human Parainfluenza virus 4 (HPIV4), Human Respiratory Syncytial Virus A (RSVA), Human Respiratory Syncytial Virus B (RSVB), Human Rhinovirus (HRV) were screened by the TaqMan low density array (TLDA) method (16) with TagMan low-density microfluidic chip technology by TaqMan^™^ Fast Advanced Master Mix (Applied Biosystems, Cat. NO: 4398986, USA) according to the manufacturer’s instructions.

### Epidemiological description and statistical analysis

On March 13, 2020, COVID-19 was declared a pandemic by WHO^1^ and on May 8, 2020, The Joint Prevention and Control Mechanism of the State Council issued a guideline on regular prevention and control of COVID-19.^2^ Accordingly, surveillance data were divided into three periods: Phase I (Jan. 1 to Mar. 1), Phase II (Mar. 1, to May 31), and Phase III (May 31 to Dec. 31). Positive rates and mean percent change for each RV were compared between phases with those of 2019.

The statistical analysis was performed using R 3.2.3 (R Core Team, R: A language and environment for statistical computing. R Foundation for Statistical Computing, Vienna, Austria). The Mantel–Haenszel Chi square test or Fisher’s exact two-tailed test was used to examine differences in discrete variable levels, with the Bonferroni corrected value of *p* of <(0.05/number of groups) indicating statistical significance.

## Results

### Characteristics of the study cases

During the two-year study period, a total of 2,744 patients were admitted the study, with 46.3% being female. The patients’ median age was 14.0 years (IQR: 4.00–43.0). The cases were divided into five groups, with 700 (25.50%), 674 (24.60%), 137 (4.99%), 768 (28.00%), and 465 (16.90%) cases, respectively. The most common symptoms of any virus positive patient observed were fever (12.21%), cough (10.39%), runny nose (6.89%), and sore throat (3.21%). Notably, the number of inpatient and outpatient visits in 2020 was significantly lower than the number in 2019. Additionally, there was a significant difference in underlying diseases observed at the time of hospitalization (Table 1).

**Table 1:** Characteristics of ARI-patients in Shanghai, China, 2019–2020.

Characteristics	Total*	2019*	2020 *	*p* value
	*N* = 2,744 (100)	*N* = 1710 (62.32)	*N* = 1,034 (37.68)	
Demography characteristics				
Gender				0.076ↆ
Female	1,271 (46.3)	815 (47.7)	456 (44.1)	
Male	1,473 (53.7)	895 (52.3)	578 (55.9)	
Age median (IQR#, years)	14.0 [4.00; 43.0]	17.0 [4.00; 44.0]	12.5 [4.00; 42.0]	0.908ↆ
Age group(years)				0.268ↆ
~5	700 (25.5)	439 (25.7)	261 (25.2)	
~15	674 (24.6)	402 (23.5)	272 (26.3)	
~24	137 (4.99)	90 (5.26)	47 (4.55)	
~60	768 (28.0)	497 (29.1)	271 (26.2)	
≥60	465 (16.9)	282 (16.5)	183 (17.7)	
Clinical characteristics				
Fever	2,125 (12.21^₸^)	1,428 (17.19^₸^)	697 (3.97^₸^)	**<0.001**ↆ
Cough	2000 (10.39)	1,380 (15.09)	620 (2.61)	**<0.001**ↆ
Runny nose	836 (6.89)	543 (9.82)	293 (2.03)	**<0.001**ↆ
Sore throat	1,120 (3.21)	686 (4.39)	434 (1.26)	**<0.001**ↆ
Expectoration	777 (2.33)	488 (3.63)	289 (0.19)	**<0.001§**
Weakness	367 (0.15)	271 (0.23)	96 (0)	**<0.001§**
Headache	307 (0.44)	261 (0.7)	46 (0)	**<0.001§**
Asthma	119 (0.07)	94 (0.12)	25 (0)	**<0.001§**
Difficulty breathing	20 (2.48)	16 (3.86)	4 (0.19)	**<0.001§**
Chest pain	48 (2.92)	30 (4.5)	18 (0.29)	0.2255**§**
Abdominal pain	39 (0.07)	21 (0.12)	18 (0)	0.656**§**
Diarrhea	31 (0.04)	14 (0.06)	17 (0)	1**§**
Hospital admission				**<0.001**ↆ
Inpatient	889 (32.4)	667 (39.0)	222 (21.5)	
Outpatient	1855 (67.6)	1,043 (61.0)	812 (78.5)	
Underlying disease	189 (6.89)	96 (5.61)	93 (8.99)	**<0.001**ↆ
Type of specimen				0.801ↆ
Nasopharyngeal swab	2016 (73.5)	1,253 (73.3)	763 (73.8)	
Sputum	728 (26.5)	457 (26.7)	271 (26.2)	

The characteristics contain demography characteristics, Clinical characteristics, hospital admission, underlying disease, and specimen. *p* values with statistical differences are shown in bold. #IQR, interquartile rang. ↆ, Mentel-Haenszel χ2 test. **§**, Fisher exact test. ₸, Positive rate of any virus infection. *Data is presented as no. (%) of patients unless otherwise indicated.

### Viral etiology detected in ARI cases

During the study period, eight different pathogens and their subtypes were detected, however, HBoV was not detected in 2020. The overall detection rate of viral infections was lower in 2020 (15.2%) compared to the same period in 2019 (34.5%), and this difference was found to be statistically significant (*p* < 0.05). Among the viruses detected, the positive rates of IFV-H1, IFV-H3, IFV-B, RSVA, HPIV1, HPIV2, and HCoVOC43 were decreased in 2020 compared to 2019. However, the detection rates of RSVB, HPIV3, HCoVNL63, and HCoV229E were significantly increased by 91.89, 24.29, 44.68 and 58.33% in 2020 (Table 2).

**Table 2:** Characteristics of ARI-patients in Shanghai, China, 2019–2020.

	Total				*P* value	Inpatient				*P* value	Outpatient				*p* value
	2019		2020			2019		2020			2019		2020		
	*n* = 1710*	%#	*n* = 1,034	%		*n* = 667	%#	*n* = 222	%		*n* = 1,043	%#	*n* = 812	%	
IFVA-H1	133	7.78	5	0.48	**<0.001**ↆ	34	5.1	1	0.45	**0.002§**	99	9.49	4	0.49	**<0.001§**
IFVA-H3	105	6.14	15	1.45	**<0.001**ↆ	18	2.7	2	0.9	0.192**§**	87	8.34	13	1.6	**<0.001**ↆ
IFVB	77	4.5	23	2.22	**0.002**ↆ	13	1.95	5	2.25	0.998ↆ	64	6.14	18	2.22	**<0.001**ↆ
HAdV	86	5.03	28	2.71	**0.003**ↆ	56	8.4	6	2.7	**0.004**ↆ	30	2.88	22	2.71	0.829ↆ
HPIV1	12	0.7	2	0.19	0.070**§**	6	0.9	0	0	0.345**§**	6	0.58	2	0.25	0.474**§**
HPIV2	8	0.47	1	0.1	0.193**§**	5	0.75	0	0	0.438**§**	3	0.29	1	0.12	0.800**§**
HPIV3	24	1.4	18	1.74	0.486ↆ	14	2.1	0	0	0.062**§**	10	0.96	18	2.22	0.027ↆ
HPIV4	8	0.47	3	0.29	0.688**§**	3	0.45	0	0	0.577**§**	5	0.48	3	0.37	0.999**§**
RSVA	28	1.64	3	0.29	**0.001§**	23	3.45	1	0.45	**0.017§**	5	0.48	2	0.25	0.667**§**
RSVB	19	1.11	22	2.13	0.033ↆ	11	1.65	7	3.15	0.270ↆ	8	0.77	15	1.85	0.037ↆ
HCoV229e	8	0.47	7	0.68	0.472ↆ	6	0.9	3	1.35	0.845**§**	2	0.19	4	0.49	0.472**§**
HCoVHKU1	11	0.64	4	0.39	0.377**§**	4	0.6	0	0	0.577**§**	7	0.67	4	0.49	0.848**§**
HCoVNL63	2	0.12	2	0.19	1.000**§**	1	0.15	0	0	1.000**§**	1	0.1	2	0.25	0.828**§**
HCoVOC43	19	1.11	5	0.48	0.087ↆ	9	1.35	2	0.9	0.863**§**	10	0.96	3	0.37	0.131**§**
HRV	33	1.93	15	1.45	0.354ↆ	12	1.8	0	0	0.094**§**	21	2.01	15	1.85	0.797ↆ
HMPV	41	2.4	5	0.48	**<0.001**ↆ	21	3.15	1	0.45	0.025**§**	20	1.92	4	0.49	**0.007§**
HBoV	7	0.41	0	0	0.095**§**	4	0.6	0	0	0.577**§**	3	0.29	0	0	0.344**§**

*P* values with statistical differences are shown in bold. #, Percentage of detected rate: Detected number of positive specimens/Total number of specimens. ↆ, Mentel-Haenszel χ^2^ test. §, Fisher exact test. *Case numbers.

### Changes of viral pathogens in hospital admission

The detection rates of IFVA-H1 (5.1 to 0.45%, *p* < 0.05), HAdV (8.4 to 2.7%, *p* < 0.05), RSVA (3.45% to.1454%, *p* < 0.05) and HMPV (3.15 to 0.45%, *p* < 0.05) in inpatients and IFVA-H1 (9.49 to 0.49%, *p* < 0.05), IFVA-H3 (8.34 to 1.6%, *p* < 0.05), IFVB (6.14 to 2.22%, *p* < 0.05) and HMPV (1.92 to 0.49%, *p* < 0.05) in outpatients in 2019 were significantly higher than those in 2020. However, the detection rates of HPIV3 (0.96 to 2.22%, *p* < 0.05) and RSVB (0.77 to 1.85%) in outpatients were increased significantly in 2020 (Table 2).

### Change of viral pathogens in gender

During the study period, significant differences were observed in the detection rate of IFVA-H1, IFVA-H3, IFVB, and HMPV for female ARI patients (8.22 to 0.44%, *p* < 0.05; 5.38 to 1.75%, *p* < 0.05; 5.15 to 2.63%, *p* < 0.05; and 2.21 to 0.66%, *p* < 0.05, respectively) and male patients (7.37 to 0.63%, *p* < 0.05; 5.92 to 1.46%, *p* < 0.05; and 2.57 to 0.42%, *p* < 0.05, respectively; Table 3).

**Table 3:** Distribution of viral etiology of the ARI-patients by age and gender, Shanghai, China, 2019–2020.

	0-4y		*p* value	5-14y		*p* value	15-24y		*p* value	25-59y		*p* value	≥60y		*p* value	female		*p* value	male		*p* value
	2019	2020		2019	2020		2019	2020		2019	2020		2019	2020		2019	2020		2019	2020	
	N*=439(%) ^#^	*n* = 261 (%)		*n* = 402 (%)	*n* = 272(%)		*n* = 90 (%)	*n* = 47(%)		*n* = 497(%)	*n* = 271(%)		*n* = 282(%)	*n* = 183(%)		*n* = 815(%)	*n* = 456(%)		*n* = 895(%)	*n* = 478(%)	
IFVA-H1	23 (5.24)	0 (0)	**<0.001** ^**§**^	11 (2.74)	2 (0.74)	0.064^**§**^	16 (17.78)	0 (0)	**0.002** ^**§**^	64 (12.88)	2 (0.74)	**<0.001** ^**§**^	19 (6.74)	1 (0.55)	**0.001** ^**§**^	67 (8.22)	2 (0.44)	**<0.001** ^**§**^	66 (7.37)	3 (0.63)	**<0.001** ^**§**^
IFVA-H3	17 (3.87)	1 (0.38)	**0.005** ^**§**^	23 (5.72)	4 (1.47)	**0.006** ^**§**^	13 (14.44)	2 (4.26)	0.07^**§**^	38 (7.65)	6 (2.21)	**0.002** ^**ↆ**^	14 (4.96)	2 (1.09)	0.025^§^	52 (6.38)	8 (1.75)	**<0.001** ^**§**^	53 (5.92)	7 (1.46)	**<0.001** ^**ↆ**^
IFVB	8 (1.82)	2 (0.77)	0.418^**§**^	17 (4.23)	12 (4.41)	0.909^**ↆ**^	11 (12.22)	2 (4.26)	0.229^**§**^	35 (7.04)	7 (2.58)	**0.009** ^**ↆ**^	6 (2.13)	0 (0)	0.117^**§**^	42 (5.15)	12 (2.63)	0.033^**ↆ**^	35 (3.91)	11 (2.3)	0.031^**ↆ**^
HAdV	32 (7.29)	6 (2.3)	**0.005** ^**ↆ**^	27 (6.72)	4 (1.47)	**0.001** ^**§**^	1 (1.11)	2 (4.26)	0.563^**§**^	17 (3.42)	9 (3.32)	0.942^**ↆ**^	9 (3.19)	7 (3.83)	0.714^**ↆ**^	28 (3.44)	8 (1.75)	0.083^**ↆ**^	58 (6.48)	20 (4.18)	0.011^**ↆ**^
HPIV1	4 (0.91)	1 (0.38)	0.735^**§**^	3 (0.75)	0 (0)	0.402^**§**^	1 (1.11)	0 (0)	1.000^**§**^	2 (0.4)	1 (0.37)	1.000^**§**^	2 (0.71)	0 (0)	0.522^**§**^	5 (0.61)	1 (0.22)	0.578^**§**^	7 (0.78)	1 (0.21)	0.234^**§**^
HPIV2	4 (0.91)	0 (0)	0.304^**§**^	2 (0.5)	0 (0)	0.518^**§**^	0 (0)	0 (0)	1.000^**§**^	0 (0)	1 (0.37)	0.353^**§**^	2 (0.71)	0 (0)	0.522^**§**^	3 (0.37)	1 (0.22)	1.000^**§**^	5 (0.56)	0 (0)	0.180^**§**^
HPIV3	12 (2.73)	4 (1.53)	0.304^**§**^	2 (0.5)	0 (0)	0.518^**§**^	0 (0)	2 (4.26)	0.116^§^	4 (0.8)	9 (3.32)	0.022^§^	6 (2.13)	3 (1.64)	0.977^**§**^	14 (1.72)	10 (2.19)	0.962^**ↆ**^	10 (1.12)	8 (1.67)	0.321^**ↆ**^
HPIV4	3 (0.68)	0 (0)	0.459^**§**^	1 (0.25)	1 (0.37)	1.000^**§**^	0 (0)	0 (0)	1.000^**§**^	3 (0.6)	0 (0)	0.499^**§**^	1 (0.35)	2 (1.09)	0.705^**§**^	2 (0.25)	2 (0.44)	0.946^**§**^	6 (0.67)	1 (0.21)	0.333^**§**^
RSVA	20 (4.56)	1 (0.38)	**0.002** ^**§**^	1 (0.25)	2 (0.74)	0.733^**§**^	0 (0)	0 (0)	1.000^**§**^	2 (0.4)	0 (0)	0.543^**§**^	5 (1.77)	0 (0)	0.177^**§**^	11 (1.35)	1 (0.22)	0.090^**§**^	17 (1.9)	2 (0.42)	**0.010** ^**§**^
RSVB	15 (3.42)	8 (3.07)	0.801^**ↆ**^	4 (1)	3 (1.1)	1.000^**§**^	0 (0)	1 (2.13)	0.343^**§**^	0 (0)	6 (2.21)	**0.004** ^**§**^	0 (0)	4 (2.19)	0.048^§^	9 (1.1)	10 (2.19)	0.125^**ↆ**^	10 (1.12)	12 (2.51)	0.139^**ↆ**^
HCoV229e	3 (0.68)	1 (0.38)	1.000^**§**^	0 (0)	1 (0.37)	0.404^**§**^	0 (0)	0 (0)	1.000^**§**^	0 (0)	2 (0.74)	0.124^**§**^	5 (1.77)	3 (1.64)	1.000^**§**^	3 (0.37)	2 (0.44)	1.000^**§**^	5 (0.56)	5 (1.05)	0.708^**ↆ**^
HCoVHKU1	5 (1.14)	3 (1.15)	1.000^**§**^	1 (0.25)	0 (0)	1.000^**§**^	0 (0)	0 (0)	1.000^**§**^	3 (0.6)	0 (0)	0.499^**§**^	2 (0.71)	1 (0.55)	1.000^**§**^	3 (0.37)	1 (0.22)	1.000^**§**^	8 (0.89)	3 (0.63)	0.613^**§**^
HCoVNL63	1 (0.23)	1 (0.38)	1.000^**§**^	1 (0.25)	0 (0)	1.000^**§**^	0 (0)	0 (0)	1.000^**§**^	0 (0)	1 (0.37)	0.353^**§**^	0 (0)	0 (0)	1.000^**§**^	2 (0.25)	1 (0.22)	1.000^**§**^	0 (0)	1 (0.21)	0.392^**§**^
HCoVOC43	9 (2.05)	2 (0.77)	0314^**§**^	4 (1)	1 (0.37)	0.636^**§**^	0 (0)	0 (0)	1.000^**§**^	4 (0.8)	1 (0.37)	0.804^**§**^	2 (0.71)	1 (0.55)	1.000^**§**^	10 (1.23)	1 (0.22)	0.122^**§**^	9 (1.01)	4 (0.84)	0.530^**§**^
HRV	8 (1.82)	2 (0.77)	0.418^**§**^	6 (1.49)	4 (1.47)	1.000^**§**^	1 (1.11)	0 (0)	1.000^**§**^	13 (2.62)	9 (3.32)	0.576^**ↆ**^	5 (1.77)	0 (0)	0.177^**§**^	15 (1.84)	8 (1.75)	0.912^**ↆ**^	18 (2.01)	7 (1.46)	0.246^**ↆ**^
HMPV	11 (2.51)	3 (1.15)	0.215^**§**^	6 (1.49)	0 (0)	0.108^**§**^	1 (1.11)	0 (0)	1.000^**§**^	13 (2.62)	1 (0.37)	0.052^**§**^	10 (3.55)	1 (0.55)	0.077^**§**^	18 (2.21)	3 (0.66)	0.038^**§**^	23 (2.57)	2 (0.42)	**0.001** ^**§**^
HBoV	4 (0.91)	0 (0)	0.304^**§**^	2 (0.5)	0 (0)	0.518^**§**^	0 (0)	0 (0)	1.000^**§**^	1 (0.2)	0 (0)	1.000^**§**^	0 (0)	0 (0)	1.000^**§**^	3 (0.37)	0 (0)	0.487^**§**^	4 (0.45)	0 (0)	0.273^**§**^

*P* values with statistical differences are shown in bold. #: Percentage of detected rate: Detected number of positive specimens/Total number of specimens. ↆ, Mentel-Haenszel χ^2^ test, **§**, Fisher exact test. *Case numbers.

### Changes of viral pathogens in age groups

During the study period, the prevalence of IFVA-H1, IFVA-H3, HAdV, and RSVA was higher in the 0–4 year age group in 2019 compared to 2020 by 100, 90.19, 68.45% and 91.67, respectively. Additionally, the 5–14 year age group had a higher prevalence of IFVA-H3, HAdV, and HMPV in 2019 than in 2020 by 74.3, 78.13 and 100%, respectively. For the 15–24 year age group, IFVA-H1was more prevalent in 2019 compared to 2020 while HPIV3 was exactly the opposite, the decrease or increase rate was 100%. Similarly, IFVA-H1, IFVA-H3, IFV-B, and HMPV were significantly more prevalent in the 25–59 year age group in 2019 by 94.25, 71.11, 63.35, and 85.88%, respectively. The older 60 years age group also saw higher prevalence of IFVA-H1, IFVA-H3, in 2019 by 91.84 and 78.02%, respectively. The differences between years were statistically significant (*p* < 0.05; Table 3).

### Changes of viral pathogens in different seasons

**Figure 1:**
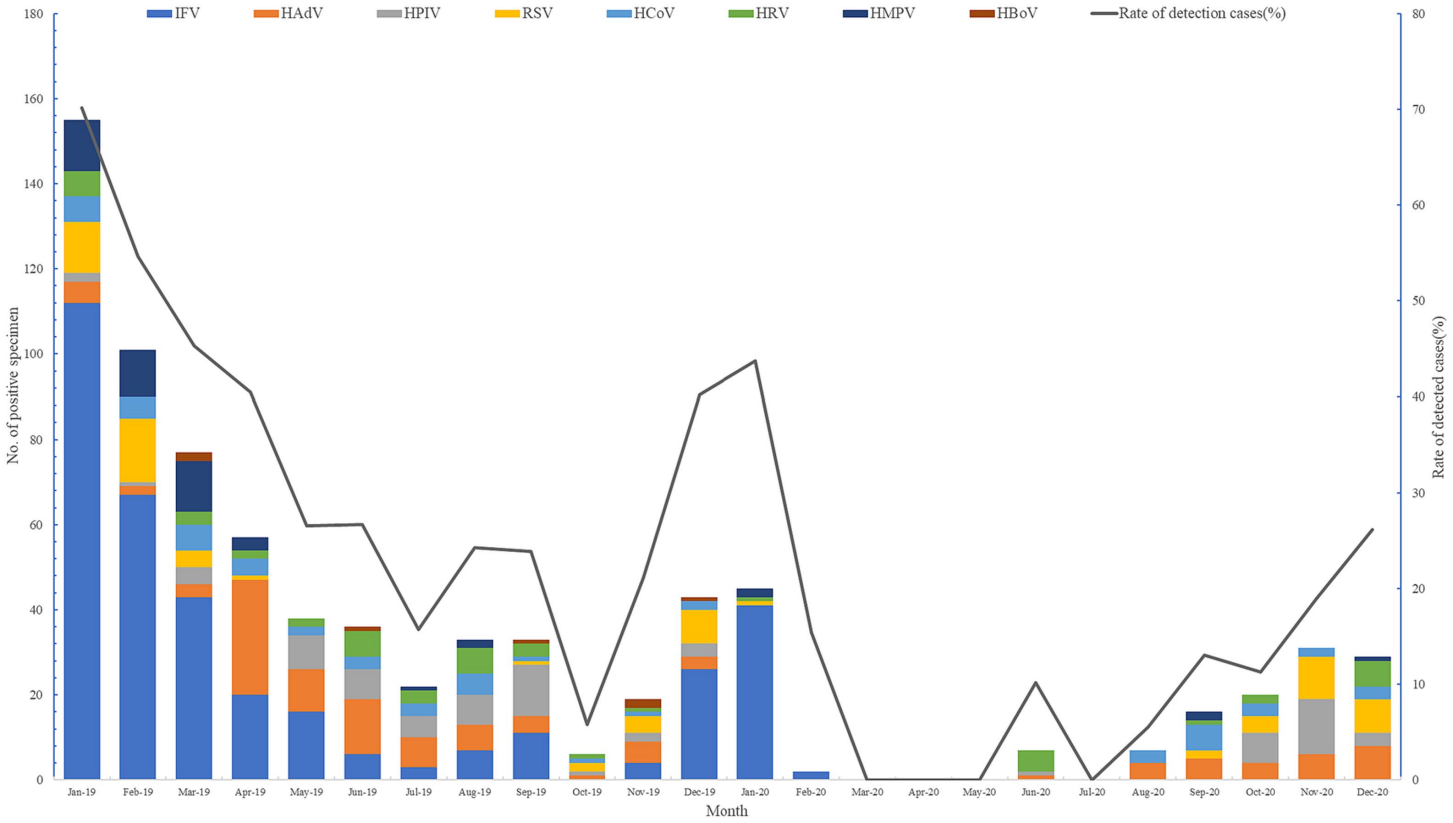
Monthly distribution of ARI-patients positive for viral etiology in Shanghai, 2019–2020.

In October of 2019, only 5.77% (6/104) of respiratory viral pathogens were screened, compared to 70.14% (155/221) at the beginning of the year. It is evident that during the winter months of 2019 and 2020, there was a significant increase in respiratory virus prevalence. From July 2020, the detection rate gradually rose until reaching its peak in December of that year. Notably, there were no respiratory viruses detected in March, April, May, or July of 2020 (see Figure 1).

### Changes of viral pathogens in different career

**Figure 2:**
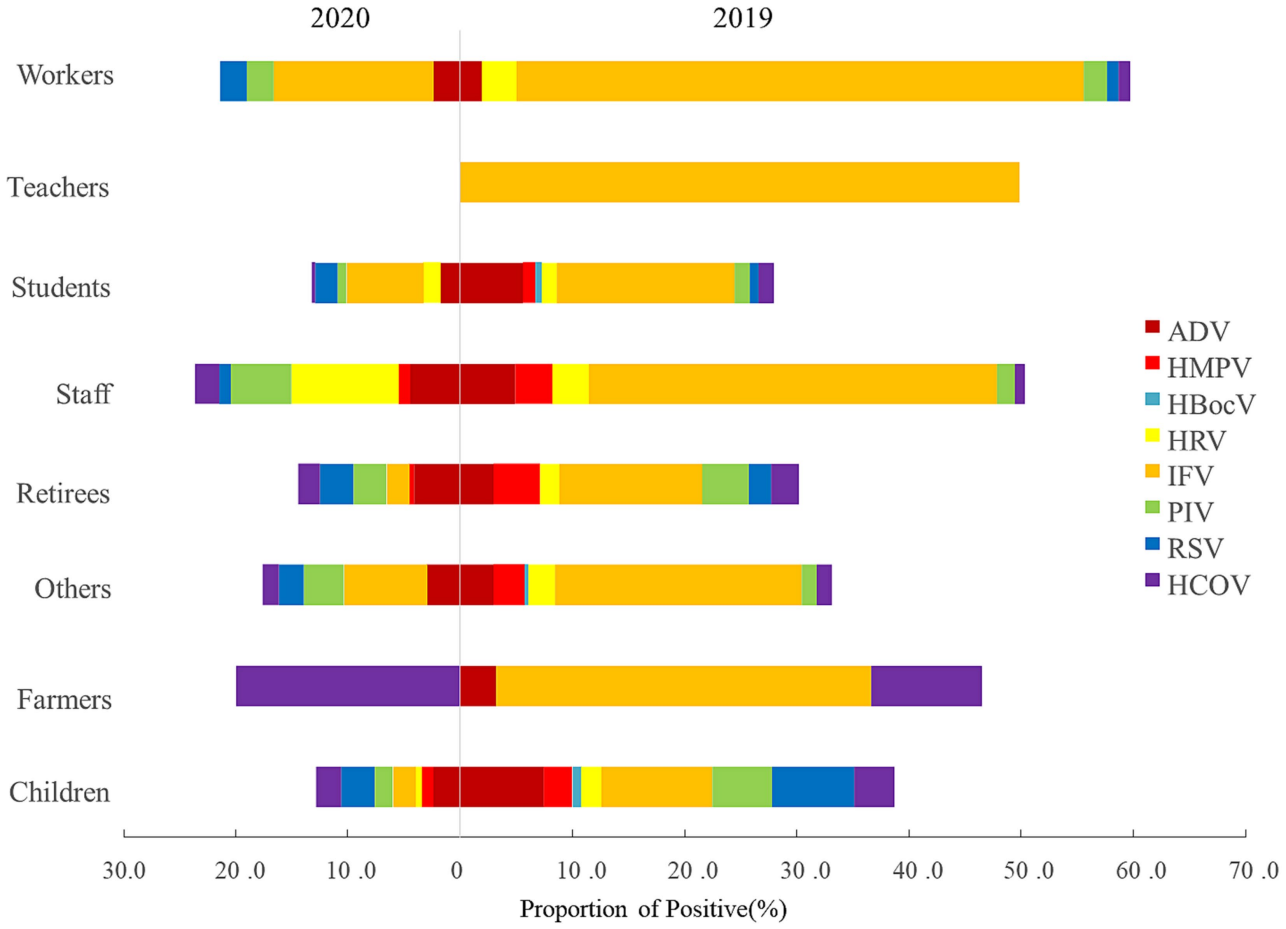
Career distribution of viral etiology in ARI-patients from 2019 to 2020, in Shanghai, China.

All 8 surveillance respiratory pathogens were detected in nursery children and students in 2019 and no RVs were detected in teachers in 2020. ARI-patients of medical personnel and farmers were infected with RSV and HCoV in 2020, respectively (Figure 2).

### Change pattern of positive rate

The detection rates of the RVs decreased from 2019 to 2020 by 22.53% (from 63.05% to 40.52), 37.89% (from 37.89 to 0%), and 9.94% (from 22.71 to 12.77%) in three phases, respectively (Table 4).

**Table 4:** Comparison of test positive rate (%) of respiratory viruses between 2019 and 2020 in Shanghai.

	Overall			Phase I*			Phase II*			Phase III*		
	2019	2020	Relative change₸	2019	2020	Relative change₸	2019	2020	Relative change₸	2019	2020	Relative change₸
IFVA-H1	7.78	0.48	−93.83%**#**	29.31	4.31	−85.30%**#**	3.08	0	−100%**#**	/		
IFVA-H3	6.14	1.45	−76.38%**#**	14.53	12.93	−11.01%	5.07	0	−100%**#**	2.71	0	−100%**#**
IFVB	4.5	2.22	−50.67%**#**	0.25	19.83	+7,832%**#**	9.25	0	−100%**#**	4	0	−100%**#**
HAdV	5.03	2.71	−46.12%**#**	1.72	0	−100%**#**	8.81	0	−100%**#**	4.59	3.22	−29.85%
HPIV1	0.7	0.19	−72.86%**#**	0.25	0	−100%**#**	0.44	0	−100%**#**	1.06	0.23	−78.30%
HPIV2	0.47	0.1	−78.72%**#**	/			0.88	0	−100%**#**	0.47	0.12	−74.47%
HPIV3	1.4	1.74	24.29%**#**	0.49	0	−100%**#**	1.32	0	−100%**#**	1.88	2.07	+10.11%
HPIV4	0.47	0.29	−38.30%	/			/			0.94	0.35	−62.77%
RSVA	1.64	0.29	−82.32%**#**	5.67	0	−100%**#**	0.44	0	−100%**#**	0.35	0.35	0%
RSVB	1.11	2.13	+1.89%**#**	0.99	0.86	−13.13%	0.66	0	−100%**#**	1.41	2.42	+71.63%
HCoV229e	0.47	0.68	+44.68%	/			0.22	0	−100%**#**	0.82	0.81	−1.22%
HCoVHKU1	0.64	0.39	−39.06%	0.74	0	−100%**#**	1.32	0	−100%**#**	0.24	0.46	+91.67%
HCoVNL63	0.12	0.19	+58.33%	0.49	0	−100%**#**	/			0	0.23	0
HCoVOC43	1.11	0.48	−56.76%	1.48	0	−100%**#**	1.1	0	−100%**#**	0.94	0.58	−38.30%
HRV	1.93	1.45	−24.87%	1.48	0.86	−41.89%	1.54	0	−100%**#**	2.35	1.61	−31.49%
HMPV	2.4	0.48	−80%**#**	5.67	1.72	−69.66%	3.3	0	−100%**#**	0.35	0.35	0%
HBoV	0.41	0	−100%**#**	/			0.44	0	−100%**#**	0.59	0	−100%**#**

*Phase I: from 1^st^ Jan. to 28^th^ Feb.; Phase II: from 1^st^ Mar. to 31^st^ May.; Phase III: from 1^st^ Jan. to 31^st^ Dec. #Statistically significant changes (value of *p*<0.05, Mentel-Haenszel chi-square test or Fisher’s exact with two-tailed). **₸**, Calculated by $\frac{\left(PR2020-PR2019\right)}{PR2019}*100\%$, PR 2019: positive rate in 2019, PR2020: positive rate in 2020.

Significant changes were identified in test-positive rates for IFV-H1, IFV-H3, IFVB, HAdV, HPIV1, HPIV2, HPIV3, RSVA, RSVB, HBoV and HMPV from 2019 to 2020. The largest decline in annual cumulative positive rates was observed by HBoV (100, 0.41 to 0%), followed by IFVA-H1 (93.83, 7.78 to 0.48%), RSVB (82.32, 1.64 to 0.29%) and HMPV (80, 2.4 to 0.48%). However, the annual cumulative positive rates for RSVB, HCoVNL63, and HCoV229e increased by 91.89% (1.11 to 2.13%), 58.33% (0.12 to 0.19%), and 44.68% (0.47 to 0.68%), respectively (Table 4).

Change pattern in RV detection rates varied significantly in three phases. In Phase I, the results showed that positive rates of HAdV (100%), HPIV1 (100%), HPIV2 (100%), HPIV3 (100%), RSVA (100%), HCoVHKU1 (100%), HCoVNL63 (100%), HCoVOC43 (100%), and IFVA-H1 (85.30%) decreased by more than 85%. The relative change in IFVB’s positive rate was the only increase in this phase and the change ratio was 7,832%. In Phase II, dramatic reductions in RV detection rates were observed and no RV detected in this phase in 2020. In Phase III, the positive rates for IFVA-H3 (100%), IFVB (100%), HBoV (100%) still decreased dramatically. However, positive rates for HCoVHKU1, RSVB and HPIV3 continued to increase, with percentage changes of 91.67, 71.63 and 10.11%, respectively (Table 4).

The detection rates of respiratory viruses decreased across three phases, representing a 22.53, 37.89, and 9.94% reduction. Notably, the annual cumulative positive rates of HBoV, IFVA-H1, RSVB, and HMPV all underwent substantial changes. Some viruses demonstrated an increase in the detection-positive rate, including RSVB, HCoVNL63, and HCoV229e.

## Discussion

To combat the COVID-19 pandemic, the Wuhan government implemented measures such as closing entertainment venues, suspending indoor public transport, and banning public gatherings (17). Subsequently, non-pharmaceutical interventions (NPIs) such as limiting social gatherings, wearing masks, practicing hand hygiene, and postponing the spring 2020 semester in primary and secondary schools were put in place to prevent the spread of SARS-CoV-2. There were no effective vaccines available globally at the time, so these NPIs were implemented to mitigate the spread of the virus (13). These interventions also had an impact on other respiratory viruses, including influenza, which was the most significant viral pathogen in ARI cases in Shanghai (14, 15).

This study analyzed the prevalence of respiratory viruses causing acute respiratory infections (ARIs) in Shanghai from 2019 to 2020. Results showed no significant differences in demographic characteristics among ARI patients in 2019 and 2020 (Table 1). Samples of ARI patients were detected by the TaqMan Low Density Array (TLDA) method from January 2019 to December 2020, despite the fact that ARI surveillance in Shanghai began in 2012 (15). This reflects the real impact of non-pharmacological interventions (NPIs) on the prevalence of respiratory viral pathogens in ARI patients.

In light of the directive to remain confined from March 1st to May 30th, 2020, the quantity of specimen collection for ARI cases decreased, which caused the decrease of the respiratory viral pathogens. Although it remains possible that ARIs were present in Shanghai during this period, this measure successfully suppressed the occurrence of respiratory viral pathogens. Correspondingly, a reduction in the number of ARI cases recorded through the surveillance system occurred, decreasing from 1,710 to 1,034, alongside a decrease in the percentage of detection of all viral pathogens, from 34.5 to 15.2%. Notably, the prevalence of ARI cases recorded was comparable to that of 2011–2015 in Shanghai (14, 15), 2016–2019 in Rome (17), and 2019–2020 in Canada (18).

Our study indicates that there are no significant differences in the demographics of ARI patients monitored over 2 years, as shown in Table 1. However, the implementation of NPIs has led to a decline in both outpatient and inpatient visits due to ARI, with the modes of transmission, including droplets, aerosols, and physical contact, being the same as those observed in COVID-19 cases, which were first detected in Shanghai after March 1, 2020. The above pattern has also been observed in respiratory tract infections in other countries, including Japan (19), Germany (20), and the United States (21).

Patients infected with IFVA had clinical symptoms such as fever and chills (22). Generally, the clinical symptoms of RVs’ infection with ARI are mild and should always be ignored. It was surprising to find that the incidence of most clinical features of ARI decreased significantly in our study. Future studies should investigate whether NPIs could relieve infection symptoms. Numerous previous studies (23–27) have shown a decrease in seasonal influenza cases in Europe, Canada, New Zealand, Japan, and the United States in late 2020. Our study indicates that IFVA incidence declined significantly during phase I and phase II, indicating that the prevalence of IFVA in early 2020 may be ending. The relative surge in IFVB cases was noteworthy, with a 7,832% increase observed in phase I, suggesting that IFVB prevalence in Shanghai began in late 2020. However, with the implementation of NPIs, the incidence and positive rates of IFVB became zero in phase II and phase III, effectively preventing the spread of the virus.

Studies in children about ADV infection showed that ADV positive rates in 2020 were significantly lower than the same period in 2019 in Hangzhou (28), Shenzhen (29), China. Our results also showed that ADV was the main RV that infected children under 14 years of age other than IFV, and ADV detection rates were much lower than in 2020.

At the beginning of 2020, a significant decline in the detection rate of RSVA was observed among children below the age of four, which is consistent with findings reported by Australian researchers (30). Furthermore, there was no notable increase in the winter season of 2020, as RSVA has consistently been the leading cause of viral pneumonia in children (23). We found that the increased positive rate of RSVB in the medical personal group in the 25-59y age group, which was also a viral pathogen in our study, increased the detection rate in this age group in Phase III in 2020.

All pathogens were affected by NPI because the test positive rates for Phase II in 2020 were zero. IFVA-H1, HAdV, HPIV1, HPIV3, RSVA, HCoVHKU1, HCoVNL63, and HCoVOC43 decreased significantly and IFVB increased dramatically in phase I. IFVA-H1 and IFVB decreased significantly in phase III. However, the detection rates of IPIV3, RSVB, and HCOVHKU1 increased (Table 4).

The biological characteristics of HCoV are similar to those of SARS-CoV-2. Interestingly, the existing four HCoVs have not changed significantly, which means that the NPIs implemented to prevent the spread of SARS-CoV-2 did not prevent existing human CoVs, especially for HCoVHKU1.

Our study used the same detection methods and patient distribution did not change significantly. There are still a number of limitations and shortcomings. Firstly, the viral activity did not know as the method was based on TLDA, one kind of polymerase chain reaction (PCR), which could not distinguish whether the virus was alive or not, although Ct values can provide information on contagiousness and NPI measures could not be inferred with the detection results. Second, the COVID-19 pandemic could change the behavior of patients with respiratory infections by viral pathogens and their health-seeking behavior could change in the future. Third, the study period is only 2 years, and the small number of Phase II and Phase III samples may lead to some errors and lack of analysis. Fourth, NPIs lead to a harsh environment for respiratory viruses, which may increase the rate of viral mutation in the near future.

Understanding the circulation pattern and prevalence of respiratory viruses is crucial for designing strategies to combat infections and outbreaks. The utilization of NPIs to combat COVID-19 has resulted in behavioral and habitual changes among individuals, consequently impacting the transmission of other respiratory viruses.

## Data availability statement

The original contributions presented in the study are included in the article/supplementary material, further inquiries can be directed to the corresponding authors.

## Ethics statement

The studies involving humans were approved by Pudong Centre for Disease Control and Prevention Ethics Review Committee. The studies were conducted in accordance with the local legislation and institutional requirements. The human samples used in this study were acquired from The acute respiratory surveillance in Pudong New Area, Shanghai, which began in 2010. Written informed consent for participation was not required from the participants or the participants’ legal guardians/next of kin in accordance with the national legislation and institutional requirements.

## Author contributions

LP: Writing – original draft, Conceptualization, Funding acquisition, Data curation, Software. QC: Writing – original draft, Data curation and Software. LH: Conceptualization and Funding acquisition. YY: Investigation. YH: Investigation. QL: Investigation. WZ: Investigation. BZ: Investigation. XZ: Data curation and Software. All authors contributed to the article and approved the submitted version.

## Footnotes

^1^ https://www.who.int/director-general/speeches/detail/who-director-general-s-opening-remarks-at-the-mission-briefing-on-covid-19---13-march-2020

^2^ https://english.www.gov.cn/policies/latestreleases/202005/08/content_WS5eb54d41c6d0b3f0e9497377.html
